# Supplementary material for: Assessment of retinal and choroidal structural and microvascular changes in early diabetic retinopathy using swept-source optical coherence tomography angiography
Source: PLoS One. 2025 Aug 1;20(8):e0327770. doi: 10.1371/journal.pone.0327770 (PMC12316237; doi:10.1371/journal.pone.0327770)
Supplement: Attachment — Submitted filename: Response to Reviewers.docx [file pone.0327770.s003.docx]

3. Have the authors made all data underlying the findings in their manuscript fully available?

The [PLOS Data policy](http://www.plosone.org/static/policies.action#sharing) requires authors to make all data underlying the findings described in their manuscript fully available without restriction, with rare exception (please refer to the Data Availability Statement in the manuscript PDF file). The data should be provided as part of the manuscript or its supporting information, or deposited to a public repository. For example, in addition to summary statistics, the data points behind means, medians and variance measures should be available. If there are restrictions on publicly sharing data—e.g. participant privacy or use of data from a third party—those must be specified.

Reviewer #1: No

Reviewer #2: Yes

4. Is the manuscript presented in an intelligible fashion and written in standard English?

PLOS ONE does not copyedit accepted manuscripts, so the language in submitted articles must be clear, correct, and unambiguous. Any typographical or grammatical errors should be corrected at revision, so please note any specific errors here.

Reviewer #1: Yes

Reviewer #2: Yes

5. Review Comments to the Author

Please use the space provided to explain your answers to the questions above. You may also include additional comments for the author, including concerns about dual publication, research ethics, or publication ethics. (Please upload your review as an attachment if it exceeds 20,000 characters)

Reviewer #1: Recent studies have investigated structural and microvascular alterations in the posterior segment of the eye across various stages of DR. Given the clinical importance of this topic, the current study has the potential to extend existing findings in the literature. The use of SS-OCTA, with its superior imaging resolution and ability to capture multiple retinal layers compared to conventional OCTA, further enhances the value of this work. However, several important concerns remain that require further clarification and elaboration.

Scientific Writing:
The manuscript would benefit from thorough proofreading to correct minor grammatical errors.
We appreciate the suggestion to replace "vascular complexes" with the more widely accepted term "capillary plexus" to avoid ambiguity. "P<0.05" has been uniformly formatted as "P < 0.05" throughout the manuscript. Redundant adverbs (e.g., "greatly lower") have been removed to enhance conciseness, with such instances revised to "significantly reduced" for more precise scientific expression.

Title:
Incorporating the evaluation of microvascular and choroidal elements might improve the title's clarity.
We appreciate the suggestions and have revised the title as follows: "Swept-source optical coherence tomography angiography assessment of microvascular and choroidal structural parameter changes in the macular and peripapillary regions in early diabetic retinopathy". The addition of "microvascular and choroidal" clarifies the assessment targets, improving the title's precision.

Abstract:
The methodology section of the abstract does not accurately summarize the study design. A concise and complete description should be provided, including the retrospective nature of the study, the imaging modality used, the classification of participant groups, and the main statistical approaches.
We thank the reviewers for their recommendations regarding the Methods section. The study design (retrospective analysis), subgroup details (mild/moderate/severe NPDR), and statistical methods (ANOVA and Kruskal-Wallis tests) have been incorporated. Redundant descriptions were removed to enhance conciseness while ensuring methodological completeness.

Methodology:
• The inclusion of images with a signal strength ≥4 may introduce inaccuracies in quantitative analysis. Many similar studies exclude images with a quality score below 6. The authors should justify this threshold and, if possible, conduct a sensitivity analysis to evaluate whether excluding lower-quality images would affect the results.
As suggested, the signal intensity threshold has been adjusted from ≥4 to ≥6, with supporting literature cited. Additionally, sensitivity analysis results have been included to strengthen methodological rigor.

• Conventionally, the perifoveal area is defined as the annular region between 3 mm and 6 mm from the foveal center, while 1–3 mm typically denotes the parafovea. Using 1–6 mm to define the perifoveal area deviates from standard anatomical definitions and could cause misinterpretation. The authors should either justify this deviation or revise their terminology to align with established standards.
We have corrected the definition of "perifoveal area" by adopting standard terminology: Parafovea (1–3 mm); Perifovea (3–6 mm). The previous nonstandard description ("1–6 mm as perifoveal area") has been removed to ensure terminological accuracy.

• A detailed explanation of how the CVI was calculated is essential. The authors should indicate whether they followed the method described by Sonada et al. or applied a modified protocol. Additionally, the region of interest used should be explicitly defined, particularly detailing the distance from the fovea that was selected. Moreover, reporting the luminal area, stromal area, and total choroidal area would add depth and transparency to the analysis.
Thank you for the suggestion. The calculation method for CVI has been clarified (as referenced by Sonada et al.), and additional parameters such as cavity area and stromal area have been included. The definition of the ROI has been specified as within 1 mm from the foveal center.

• The manuscript should clearly specify where VD was measured within the superficial and deep vascular complexes—whether in the total area, fovea, parafovea, or perifovea.
Thank you for the suggestion. The first paragraph of Section 2.3 of the manuscript has been revised accordingly.

• The authors refer to the “superficial and deep vascular complex”, rather than the more widely used “superficial and deep capillary plexus”. They should clarify whether this terminology reflects specific segmentation settings from the imaging software or manufacturer conventions. A consistent use of terms and clear definitions of layer boundaries are necessary to enhance clarity and reproducibility.
Thank you for the suggestion. The measurement region for VD has been clarified (full retina, parafoveal/perifoveal regions). The term "vascular complex" has been explained as the device's layering setting, and it is synonymous with the term "capillary plexus."

Results
• All statistical comparisons should be reported with corresponding effect sizes. Mean and SD for normally distributed data, or median and IQR for non-normally distributed data.
Thank you for the suggestion. The results section has been updated to include the mean difference, standard deviation, and effect size (Cohen's d). The format for non-normally distributed data has been clearly indicated as median \[IQR], along with the corresponding statistical test used.

Discussion
• The discussion could be strengthened by elaborating on how the findings may inform clinical decision-making. For instance, can specific SS-OCTA parameters or thresholds be used to predict disease progression or guide earlier intervention strategies?
Thank you for the suggestion. A new paragraph has been added to the discussion, focusing on clinical decision-making recommendations and threshold guidance. Based on effect sizes (HR, OR), actionable clinical thresholds have been proposed. Additionally, the follow-up frequency and intervention measures have been clearly defined.

• The limitations section should include a discussion on the retrospective nature of the study and potential confounding factors (e.g., duration of diabetes, level of glycemic control) that may have influenced the results.
Thank you for the suggestion. A new section has been added to the discussion, addressing retrospective dye and confounding factor analysis. The limitations of retrospective studies, including selection bias and confounding factors, have been emphasized. Specific data (e.g., duration of diabetes) have been cited to illustrate potential impacts.

Figures and Tables
• Consider adding a summary table that lists the key parameters.

Reviewer #2: First of all, I would like to thank the authors for their efforts in this manuscript. This manuscript is titled Adoption of Swept-source Optical Coherence Tomography Angiography in Measuring Retinal Structural Parameter Changes in the Macula and Peripapillary Area in Early Diabetic Retinopathy. Although the overall quality of this manuscript is good, the following comments are needed to improve its overall quality:
1. Please specify what is the study design? Retrospective cohort or case-control? How was the matching between groups done?
Thank you for the suggestion. In response, the study design in Section 2.1 has been explicitly stated as a "retrospective cohort study." Additionally, the inter-group matching method has been supplemented, including stratified sampling based on age, sex, and hypertension status.

2. The abstract should include the number of participants and the important results in quantitative terms. The exact p-value along with the confidence interval is valuable.
Thank you for the suggestion. In response, the abstract has been updated to include the number of participants (208 diabetic cases and 51 controls). Quantitative results, including mean difference, confidence intervals, and trend tests, have also been added.

3. The significance of the study should be stated in the introduction.
Thank you for the suggestion. In response, the innovation of this study has been highlighted at the end of the introduction, emphasizing its clinical significance in guiding early intervention and addressing the limitations of traditional diagnostic methods.

4. The limitations of the study should be stated in the discussion. It is also recommended that the researchers provide directions for future studies.
In response to the suggestion, the limitations of this study have been added to the discussion section.

5. How was the sample size assessed?
Thank you for the suggestion. In response, the sample size calculation basis (effect size, α, β) has been clearly stated in Section 2.1. Additionally, the adjustment for the loss to follow-up rate has been explained.

6. It is recommended that this study be organized based on the STROBE reporting checklist and the completed checklist be submitted as an appendix.
Thank you for the suggestion. In response, this study strictly adheres to the STROBE (Strengthening the Reporting of Observational Studies in Epidemiology) guidelines. The complete checklist is provided in Supplementary Material Table S2, and the STROBE checklist has been submitted in the appendix.

7. It is recommended that the STROBE flow diagram be included in the results.
Thank you for the suggestion. In response, a flowchart has been added to Section 3.1 of the results.

8. What do a, ab, etc. mean in the figures? This should be mentioned in the figure caption.
Regarding the figures, the meaning of multiple comparison symbols (a, b, c, d) has been clearly specified in the figure caption.

9. In the method, it is necessary to carefully specify the inclusion criteria and exclusion criteria.

Additionally, in the methods section, the inclusion and exclusion criteria have been carefully detailed, including specific age ranges, treatment history, and examples of excluded disease types (e.g., glaucoma, macular holes).
