# Supplementary material for: Assessment of retinal and choroidal structural and microvascular changes in early diabetic retinopathy using swept-source optical coherence tomography angiography
Source: PLoS One. 2025 Aug 1;20(8):e0327770. doi: 10.1371/journal.pone.0327770 (PMC12316237; doi:10.1371/journal.pone.0327770)
Supplement: Attachment — Submitted filename: Response_to_Reviewers_auresp_2.docx [file pone.0327770.s004.docx]

6. Review Comments to the Author

Please use the space provided to explain your answers to the questions above. You may also include additional comments for the author, including concerns about dual publication, research ethics, or publication ethics. (Please upload your review as an attachment if it exceeds 20,000 characters)

Reviewer #1: I appreciate the authors’ revisions and their efforts to improve the manuscript.
However, several key issues remain.
• The title still could benefit from improved clarity; I suggest: “Assessment of Retinal and Choroidal Structural and Microvascular Changes in Early Diabetic Retinopathy Using Swept-Source Optical Coherence Tomography Angiography.”
Thank you for your suggestions. In accordance with your recommendation, the title of the manuscript has been revised to: “Assessment of Retinal and Choroidal Structural and Microvascular Changes in Early Diabetic Retinopathy Using Swept-Source Optical Coherence Tomography Angiography.”

• Regarding the revised signal strength threshold, it is unclear whether the data were reanalyzed accordingly or if the previous threshold was a typographical error; this should be clarified.
Thank you for your suggestions. In accordance with your recommendation, the content in section 2.2 of the manuscript has been supplemented as follows: This threshold (≥6) was consistently applied during initial data acquisition and processing throughout the study. All analyses were performed based on this predefined criterion without retrospective reanalysis.

• Additionally, the revised results still emphasize mean differences rather than reporting group-wise means and SDs. All comparisons should present raw summary statistics (mean ± SD or median [IQR]).
Thank you for your suggestions. In accordance with your recommendation, a description of the raw data for each group has been added to the Results section of the manuscript.

• Finally, the limitations section includes a redundant sentence that should be removed for clarity: “This study used SS-OCTA technology to analyze in detail the changes in retinal structural parameters in macula and optic disc regions in patients with NDR and various stages of NPDR.”

Thank you for your suggestions. In accordance with your recommendation, the redundant statements have been removed from the Discussion section of the manuscript.

Reviewer #2: Thanks to the authors for their efforts in addressing the peer review comments. The only issue that I think still remains is the STROBE flow diagram, where the number 51 is derived from 208 (which seems wrong). It seems that a two-armed figure should have been used in this study.

Thank you for your suggestions. In accordance with your recommendation, the flowchart in the manuscript has been revised.
